# Drylands Under Pressure: Responses of Insect Density to Land-Use Change in a Tropical Desert

**DOI:** 10.3390/insects16101043

**Published:** 2025-10-11

**Authors:** Anshuman Pati, Indranil Paul, Sutirtha Dutta

**Affiliations:** 1 Wildlife Institute of India, P.O. Box 18, Chandrabani, Dehradun 248001, Uttarakhand, India; 2 Academy of Scientific and Innovative Research (AcSIR), Ghaziabad 201002, Uttar Pradesh, India; 3 IUCN Grasshopper Specialist Group, 1196 Gland, Switzerland; 4 Forest Research Institute (Deemed to be) University, Dehradun 248195, Uttarakhand, India; 29indranil@gmail.com

**Keywords:** land-use, insect, grazing, orthoptera, conservation, Thar Desert

## Abstract

Habitat alteration due to agricultural expansion and heavy livestock grazing is a major threat for open natural ecosystems (ONEs). Within the Indian Thar Desert, such land-use transformations are altering native grassland habitats, with consequential effects on insect communities that perform vital ecological functions and support higher trophic levels. Between 2020 and 2022, we surveyed a 641 km^2^ area, using belt transect and visual detection methods, to quantify insect densities at the order level across different seasons. Linear mixed-effect (LME) models revealed that the orthopteran insect densities, primarily grasshoppers, were significantly higher in grasslands compared to agriculture and barren lands and were lower in the presence of livestock grazing. Orthopteran densities were higher and showed strong seasonal dependencies, likely driven by rainfall-mediated vegetation growth during monsoons. Intense grazing and agricultural expansion reduced vegetation biomass and resource availability, which affected the insect populations negatively. These research findings underscore the urgent need to implement ecologically sensitive land management practices, including sustainable grazing regimes and grassland conservation, to maintain insect biodiversity and the broader ecological network. Given the role of insects in ecosystem functioning and their importance to conservation dependent species of, such as the critically endangered Great Indian Bustard (*Ardeotis nigriceps*), these findings underscore the ecological significance of preserving native grassland habitats in the Thar Desert landscape.

## Simple Summary

Responses of insects to land-use transformation and grazing in the Indian Thar Desert were examined across 641 km^2^ area from 2020 to 2022. Systematic belt-transect surveys showed significant variation in insect densities across habitats and seasons. Grasslands harboured significantly greater densities in monsoon, dominated by Orthoptera, compared to agriculture, barren, or scrub habitats, while livestock grazing further reduced Orthopteran populations. Coleoptera and other taxa displaying weaker habitat affinities. Insect densities exhibited sharp seasonality, surging during monsoon and collapsing in summers. Even low-intensity cultivation and unmanaged grazing were sufficient to diminish insect populations and disrupt trophic networks. Conservation of structurally diverse grasslands, supported by low-intensity grazing regimes, is essential for maintaining insect biodiversity and ensuring prey availability for higher trophic levels, particularly the critically endangered Great Indian Bustard.

## 1. Introduction

Habitat loss is a significant conservation challenge of current times, affecting population distribution and the abundance of many species [1,2,3]. The modification of natural habitats for agriculture is the most frequent form of global land-use change [4,5,6]. Agricultural activities reduce available habitat and resources (food, shelter, and mates) for wildlife populations and change the landscape structure and configuration, leading to several ecosystem-level consequences [7,8] such as impaired plant dispersal and animal movements, population collapse, loss of trophic interactions, and regional climate change [9,10]. Open Natural Ecosystems (ONEs) are perhaps most threatened by this change, due to their arability, accessibility, and prolonged history of human habitation [11]. Herein, conversion of grasslands to croplands affects insect communities in multiple ways [12,13].

During the past 140 years, India has experienced remarkable land-use and land-cover changes (LULCC), including deforestation, cropland changes, and urban expansion [14,15]. Additionally, excessive grazing by free-ranging livestock has caused major degradation of vegetation cover in arid regions [16,17]. About 11% of Earth’s land surface is used for agriculture and 30% for grazing [18]. The Land Degradation Assessment in Drylands concluded that ~16% of rangelands are undergoing degradation [19,20]. In India, a heavily populated and biodiversity-rich region, grasslands are largely ignored in conservation discourse and in post-colonial land policies, despite the multitude of ecosystem services, dependency of human livelihoods, and high biodiversity values of these ecosystems [21,22]. India’s ONEs now thrive only in pockets, the largest being the arid zone of the Thar Desert [23,24]. Grassland patches in the Thar Desert offer various resources for specialised wildlife species and subsistence livelihoods [21,25]. Numerous insects are found to rely on desert grasslands for food, shelter, and egg-laying sites [26].

Terrestrial insects play vital ecosystem roles as detritivores, scavengers, pollinators, and biological control agents [27]. However, insect populations are declining at an alarming rate in most parts of the world [28,29,30], garnering global attention [31]. The global populations of 33% of insect communities have shown declining trends with strong variation among insect orders: Orthoptera followed by Coleoptera, Hymenoptera, Lepidoptera, and Odonata [32]. Such declines pose serious problems in insect-mediated ecosystem functioning and populations of insectivorous animals [33]. Insect communities are often associated with specific habitats [34] and can be ideal bioindicators of the habitat [35].

Deserts are found on almost all the continents and experience extremely hot summers, cold winters, and deficient rainfall [36]. In spite of its extreme conditions, the Thar Desert holds a unique biodiversity that is adapted to its xeric environment and is facing escalated land-use conversion in recent times, stemming from its high human density (relative to other deserts). Due to its erratic and low rainfall, with a high inter-annual variability that influences the crop yield [37], only one rainfed crop is traditionally grown during good rainfall years in this region [38]. However, the agricultural cover has expanded over the years due to expanding human habitation, availability of surface water, and the advent of mechanised farming, resulting in a concomitant intensification of grazing pressure on the shrinking rangelands. On the other hand, the region has gained significant conservation attention in recent years because of the single viable population of the Critically Endangered Great Indian Bustard *Ardeotis nigriceps* (GIB), because of which some grassland patches are being protected from land-use conversion [39]. The persistence of this wide-ranging species depends on large contiguous habitats, as fragmented habitats or small protected areas are insufficient to support its ecological requirements [25]. This flagship species is an insectivorous omnivore, and understanding how ongoing land-use conversion impacts insect communities is vital for its conservation. Insect abundance is expected to vary seasonally, depending on temperature and rainfall regimes [40]. The insect diversity and assemblage of a region are often related to the vegetation composition and habitat structure [41]. Several insects have seasonally tuned life cycles that are highly synchronised with abiotic environmental changes. This is particularly relevant for desert landscapes that undergo high variability in vegetation biomass, structure, and composition between seasons. Ecological studies on insects and their responses to land use are extremely scarce for this region. This study examines how the density of various insect orders responds to land covers and uses, including agriculture vs. natural vegetation with and without livestock grazing, across seasons in the Thar Desert.

## 2. Materials and Methods

### 2.1. Study Area

**Figure 1:**
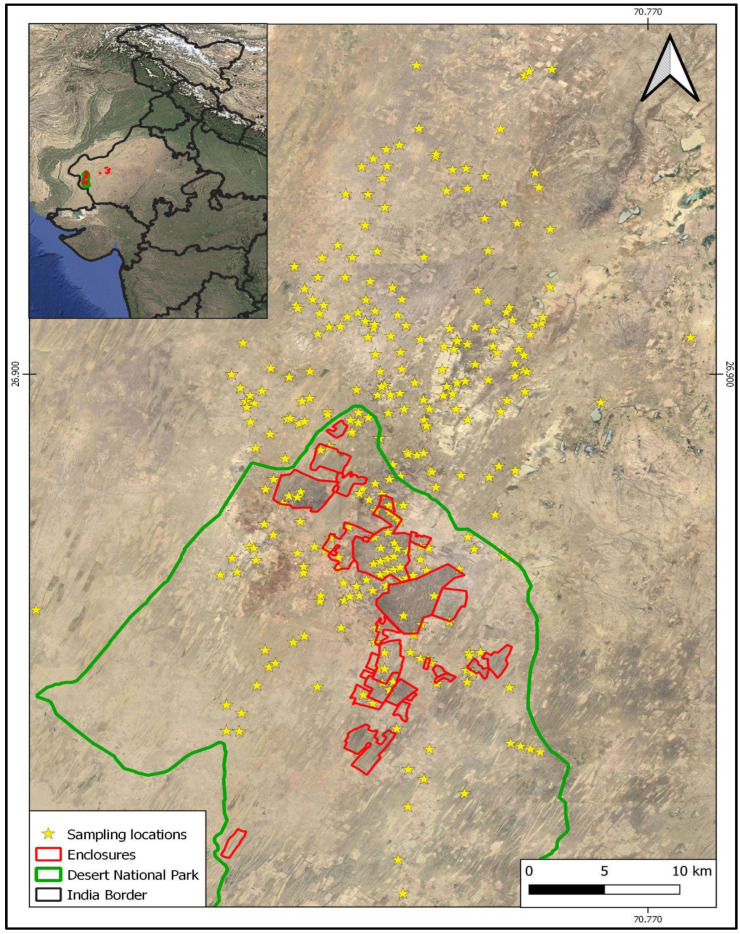
Map of the study area, Desert National Park, and associated areas.

The Indian Thar Desert largely comprises vegetated and bare sand dunes, grasslands dotted with shrubs and trees, and sand/soil substrate [42]. It occupies a ~385,000 km^2^ area and about 9% of the country’s land surface [43]. The Desert National Park (DNP) and its satellite enclosures constitute one of the largest and only Protected Areas (PAs) of the Thar region. This PA encompasses 3162 km^2^ in the Jaisalmer and Barmer Districts of Rajasthan. The climate is characterised by hot, dry summers; cold, dry winters; low and erratic annual rainfall; and high wind velocity. Some areas in the PA are protected by fencing and managed for conservation-dependent species, such as the Great Indian Bustard (Figure 1). Areas outside these grassland reserves have unrestricted land uses and human activities. There are several small agro-pastoral villages within the DNP. The study was conducted in a 641 km^2^ area in and around the DNP, covering dominant land covers and uses from 2020 to 2022 in the monsoon (July–September), post-monsoon (October–November), winter (December–February), and summer (April–June) seasons.

### 2.2. Field Sampling

Random sampling points (n = 554) were systematically generated across the study area (641 km^2^) using the programme QGIS (ver 3.8.2). At each point, a belt transect of a 50*2 m^2^ dimension was laid in a randomly assigned direction. Transects were walked at a slow and steady speed during fixed temporal windows between 06:30–09:30 and 16:00–18:30 h, to maximise the detection of diurnally active taxa, and minimise noise. All insects, especially Orthoptera, Coleoptera, and others (Hemiptera, Hymenoptera, Odonata, Neuroptera, and Diptera) encountered visually along transects, were recorded and identified up to the order level [44].

In this landscape, the sparse vegetation allowed for near-perfect detection of insects within the transect strip, thus allowing for an unbiased detection of major insect orders, using a fairly unintrusive technique compared to other methods of sampling. Nocturnal and/or cryptic insects will be missed in this visual detection method. For capturing these insects and obtaining a more comprehensive assessment of insect diversity, methods such as pit falls or sweep net sampling could be used in the future; however, pooling data from such different methods is fraught with its own drawbacks, due to the varying capture coverage and detectability of each method, and was not considered here, as the study focused on diurnal insects for their relevance to the conservation of the flagship GIB and other insectivores [45].

The dominant land covers—grassland, agriculture, barren, and scrubland—were recorded within a 100 m circular plot of the sampling point in each season. The presence of livestock signs (direct observation, dung/pellet, and trails) within the plot was recorded in 1*1 m quadrats at four cardinal directions.

### 2.3. Statistical Analyses

We tested the effects of habitat and livestock grazing on the density of insect orders by modelling log-transformed counts (detections in 100 m^2^ plots) of Orthoptera, Coleoptera, and other insect taxa using linear mixed-effect models. Land cover and livestock grazing were included as fixed effects, whereas variations in the densities of insect taxa among seasons and years were accounted for as nested random effects [46]. We compared candidate models, including univariate and additive effects of these predictors, using an Information Theoretic approach and inferred parameter estimates from the least Akaike Information Criterion (AICc) models. We examined our models for a violation of assumptions using residual diagnostics. We carried out the data entry in MS Excel 2007 and statistical analysis in the programme R 4.3.1.

## 3. Results

**Figure 2:**
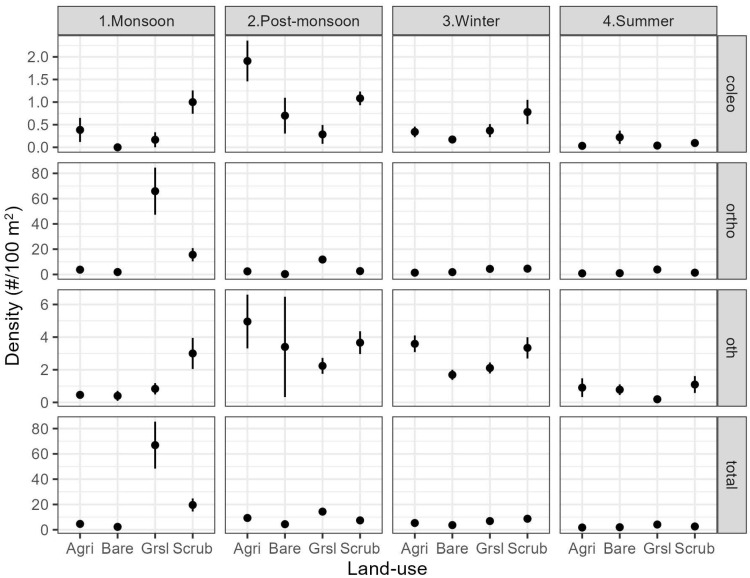
Mean (SE error bars) of insect density (ortho: Orthoptera, coleo: Coleoptera, oth: Others) against land covers (Agri: Agriculture, Bare: Barren, Grsl: Grassland, and Scrub: Scrubland) across seasons (columns) and rows (insect orders) in/around Desert National Park.

The density of insect orders varied across land covers and seasons (Figure 2). The insect density (individuals per 100 m^2^) was estimated at 65.9 ± 18.2 (Mean ± SE) (Orthoptera), 0.16 ± 0.16 (Coleoptera), 0.8 ± 0.3 (other insect orders), and 66.9 ± 18.6 (all insects) in grasslands during monsoon season, the resource-abundant season, whereas, in summer—the resource-poor season—the insect density was much lower, estimated at 3.90 ± 1.06 (Orthoptera), 0.04 ± 0.02 (Coleoptera), 0.19 ± 0.13 (other insect orders), and 4.1 ± 1.1 (all insects) (Figure 2). A comparison of candidate models supported the hypothesised effects of land cover and livestock grazing on the Orthopteran density (summed Akaike wt of best model, W = 0.78), whereas there was no effect of these variables on the Coleopteran density, and some effect of land cover, but not of livestock grazing, was observed on other insect orders (W = 0.87) (Table 1). The Orthopteran density was greater in the grassland and lower in the scrubland, agricultural, and barren areas (Table 2). The Orthopteran density reduced in the presence of livestock grazing. The density of other insects was greater in agricultural than in grassland or barren areas. The pooled abundance of insects was greatest in grasslands during the monsoon season, primarily due to high numbers of Orthopterans, and was less variable across land covers during other seasons. The model diagnosis did not show a violation of the important linearity assumption, although it was indicative of asymmetric distribution of residuals (towards positive values), implying some overdispersion for Coleopteran and other insect orders, but not for Orthoptera.

## 4. Discussion

The accelerated conversion of natural habitats into agriculture to meet growing human demands imperils grassland ecosystems, covering ~40% of the Earth’s terrestrial area, as these habitats are highly arable, easily accessible, and are under prolonged human footprint [11,47]. Grasslands in India are considered wastelands and are prioritised for economic development over nature conservation [22]. The consequent rapid land-use change has not only degraded this unique and antique habitat [48] but has also endangered many specialised wildlife that depend on it [21]. Global studies have shown adverse effects of land-use change on insects, which are key ecological indicators [18,49].

Our study found markedly higher insect densities in grasslands compared to agricultural areas. This finding aligns with patterns observed in other desert-margin ecosystems, where habitat degradation through overgrazing and agricultural expansion has led to declines in insect populations and community complexity [18,49]. The insect population in the Thar Desert is numerically dominated by Orthopterans, mainly grasshoppers. The density of grasshoppers was significantly lower in agricultural fields and other land covers compared to grasslands. Furthermore, the Orthopteran density was also lower in areas with high livestock grazing. Thus, the Orthopteran population, and therefore overall insect abundance, tends to decline in modified habitats, especially under mowing, grazing, or conversion to cropland, which reduces the habitat complexity and plant diversity [50,51].

In the Thar Desert, although agriculture is largely rainfed, the availability of water through the Indira Gandhi Nahar Project and a recent trend of higher rainfall and more surface water have collectively accelerated agricultural encroachment in natural habitats during the last few years [52,53]. This transformation has profound implications for insect conservation, particularly for taxa such as grasshoppers (Orthoptera), which are highly dependent on the vegetation structure and microclimatic stability for feeding, thermoregulation, and reproduction [50,54]. Due to their dependency on the vegetation and microclimate, they are effective bioindicators of the grassland health and management [54]. Notably, controlled livestock grazing in grasslands has been shown to maintain vegetation heterogeneity and reduce outbreaks of pest arthropods, thereby balancing conservation and agricultural objectives [55].

We also found a noticeable variation between taxa in their response to different land covers. In contrast to Orthopterans, diurnal Coleopteran insect assemblages did not show any preference for any particular land cover and did not decrease in modified (agricultural) habitats. Thus, habitat heterogeneity, where multiple natural land covers are interspersed, might boost insect diversity at order/family levels.

Moreover, the simplification of land cover led to homogenised insect assemblages, reduced temporal stability, and diminished trophic interactions [31,56]. The densities of most insect orders were generally lower in barren or sparsely vegetated areas, likely mediated through bottom–up mechanisms of food shortage [49,57]. These findings highlight the need to conserve structurally intricate and floristically assorted habitats to uphold insect biodiversity and ecological resilience.

Insect communities and their populations are sensitive to seasonal changes in habitat [58,59,60], a phenomenon that was also observed in our study. Desert ecosystems are shaped by monsoons [61,62]. Rainfall, the primary driver of vegetation growth in the desert, provides a seasonal pulse of resources for the proliferation of insects [63]. Our study showed that grasshopper numbers increased profusely during the monsoon season. A high insect population coincides with the breeding period of other higher taxa, such as many grassland insectivore birds, including the critically endangered *Ardeotis nigriceps* [64]. Breeding cycles of desert birds are shaped by food resources, mainly grasshoppers [64], further highlighting their importance in maintaining the ecological integrity of desert ecosystems.

To conclude, our study shows that even less-intensive, low-yield agricultural practices, without inorganic inputs and mechanisation, can reduce the grasshopper and, therefore, overall insect abundance remarkably, compared to unexploited natural grasslands, highlighting the need for strategic grassland management to safeguard insect communities in tropical desert ecosystems. Also, insect-order-level analyses offer a valuable macroecological lens, revealing broad habitat associations and seasonal trends that inform baseline conservation priorities. Insect populations are crashing worldwide [65], and even low-intensity habitat modifications can affect their densities, as revealed by our study, which corroborates the ongoing decline in higher taxa populations, such as insectivorous birds of grassland ecosystems [66]. Understanding Orthoptera–vegetation interactions, grazing effects, and climatic influences requires long-term, multi-seasonal research. The conservation and restoration of grasslands are vital not only for sustaining insect communities but also for preserving the broader ecological network, including critically endangered species, such as the Great Indian Bustard, that depend on insects as the prime food resource for their energy-demanding breeding activity [67].

## Figures and Tables

**Table 1:** Ranking of candidate mixed-effect models based on Akaike Information Criteria (AICc), explaining density patterns of insect orders as fixed effects of land cover (hab) and/or livestock grazing (grz), along with random effects of season nested within years, in/around Desert National Park. Other summary statistics for models include degrees of freedom (K), log-likelihood value (logL), difference in AICc from least AICc model (delta), and Akaike weight (Ak. wt).

Model	K	logL	AICc	delta	Ak. wt
(Orthoptera)					
Y ~ hab + grz + (1|year/season)	8	−709.20	1434.7	0.00	0.74
Y ~ hab + (1|year/season)	7	−711.26	1436.7	2.08	0.26
Y ~ 1 + (1|year/season)	4	−765.89	1539.9	105.2	0.00
Y ~ grz + (1|year/season)	5	−765.03	1540.2	105.5	0.00
(Coleoptera)					
Y ~ 1 + (1|year/season)	4	−354.05	716.2	0.00	0.40
Y ~ grz + (1|year/season)	5	−353.19	716.5	0.32	0.34
Y ~ hab + (1|year/season)	7	−351.95	718.1	1.95	0.15
Y ~ hab + grz + (1|year/season)	8	−351.25	718.8	2.60	0.11
(Other insects)					
Y ~ hab + (1|year/season)	7	−595.71	1205.6	0.00	0.87
Y ~ hab + grz + (1|year/season)	8	−596.98	1210.2	4.59	0.09
Y ~ 1 + (1|year/season)	4	−602.00	1212.1	6.44	0.04
Y ~ grz + (1|year/season)	5	−603.16	1216.4	10.80	0.00

**Table 2:** Parameter estimates of fixed and random effects from best model (least AICc) explaining density patterns of insect orders in/around Desert National Park during.

	Random Effect			Fixed Effect				
	SD of Groups			Estimate (SE)				
Insect Order	Season: Year	Year	Residual	Intercept	Barren	Grassland	Scrubland	Livestock Grazing
Orthoptera	0.71	0.20	0.88	0.98 (0.40)	−0.27 (0.14)	0.99 (0.11)	0.25 (0.11)	−0.29 (0.11)
Coleoptera	0.18	0.09	0.46	0.21 (0.12)	-	-	-	-
Others	0.30	0.37	0.72	0.71 (0.32)	−0.34 (0.12)	−0.27 (0.09)	0.03 (0.09)	-

## Data Availability

The raw data supporting the conclusions of this article will be made available by the authors on request.
